# Endocannabinoid-Like Lipid Neuromodulators in the Regulation of Dopamine Signaling: Relevance for Drug Addiction

**DOI:** 10.3389/fnsyn.2020.588660

**Published:** 2020-12-23

**Authors:** Claudia Sagheddu, Larissa Helena Torres, Tania Marcourakis, Marco Pistis

**Affiliations:** ^1^Department of Biomedical Sciences, Division of Neuroscience and Clinical Pharmacology, University of Cagliari, Cagliari, Italy; ^2^Department of Food and Drugs, School of Pharmaceutical Sciences, Federal University of Alfenas, Alfenas, Brazil; ^3^Department of Clinical and Toxicological Analysis, School of Pharmaceutical Sciences, University of São Paulo, São Paulo, Brazil; ^4^Neuroscience Institute, National Research Council of Italy (CNR), Section of Cagliari, Cagliari, Italy

**Keywords:** *N*-acylethanolamines, endocannabinoids, dopamine neurons, peroxisome proliferator-activated receptors-α, nicotine, alcohol

## Abstract

The family of lipid neuromodulators has been rapidly growing, as the use of different -omics techniques led to the discovery of a large number of naturally occurring *N-*acylethanolamines (NAEs) and *N-acyl* amino acids belonging to the complex lipid signaling system termed endocannabinoidome. These molecules exert a variety of biological activities in the central nervous system, as they modulate physiological processes in neurons and glial cells and are involved in the pathophysiology of neurological and psychiatric disorders. Their effects on dopamine cells have attracted attention, as dysfunctions of dopamine systems characterize a range of psychiatric disorders, i.e., schizophrenia and substance use disorders (SUD). While canonical endocannabinoids are known to regulate excitatory and inhibitory synaptic inputs impinging on dopamine cells and modulate several dopamine-mediated behaviors, such as reward and addiction, the effects of other lipid neuromodulators are far less clear. Here, we review the emerging role of endocannabinoid-like neuromodulators in dopamine signaling, with a focus on non-cannabinoid *N-*acylethanolamines and their receptors. Mounting evidence suggests that these neuromodulators contribute to modulate synaptic transmission in dopamine regions and might represent a target for novel medications in alcohol and nicotine use disorder.

## Introduction

One of the most fascinating fields in contemporary neuroscience is the emergence of lipids as signaling molecules, with a multitude of compounds recognized as mediators of communication within and between neurons (Piomelli et al., 2007). Among lipid neuromodulators, research in the last two decades has been focusing on synthesis, cellular effects, and catabolism of endogenous cannabinoids (eCBs) and their CB1 and CB2 cannabinoid receptors. The first characterized eCB, *N-arachidonoylethanolamide* (anandamide, AEA; Devane et al., 1992; Table 1), is one member of the *N-*acylethanolamines’ (NAEs) family, also termed fatty acid ethanolamides. NAEs differ in the length and saturation of the hydrocarbon chain and their receptor affinity (Schmid et al., 1990; Hansen et al., 2000). Besides AEA, the saturated palmitoylethanolamide (PEA) and the monounsaturated oleoylethanolamide (OEA) have attracted attention due to their biological effects in the brain and periphery (Table 1). *N*-acyl amino acids are another related family of lipid signaling molecules in which an amino acid is covalently linked by an amide bond to the acyl moiety of a long-chain fatty acid. Among *N-acyl* amino acids, *N-acyl* glycines (particularly *N-*arachidonylglycine and *N-*oleoylglycine) are emerging as an intriguing class of neuromodulators, although largely uncharacterized so far (Bradshaw et al., 2009; Burstein, 2018; Battista et al., 2019; Table 1).

**Table 1:** Representative bioactive neural lipids, their cellular receptors, and their cellular effects on dopamine cells.

	Cellular receptors	Effects on dopamine cells
**Endocannabinoids**
2-arachidonoylglycerol (2-AG) 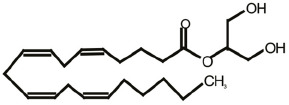	CB1 (Sugiura et al., 1995), CB2 (Mechoulam et al., 1995)	Short-term synaptic depression of glutamate and GABA inputs (Melis et al., 2004a)
Arachidonoylethanolamide (Anandamide) (a cannabinoid *N*-acylethanolamine) 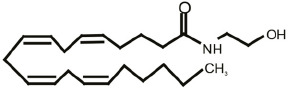	CB1 (Devane et al., 1992), CB2 (Mechoulam et al., 1995), TRPV1 (Zygmunt et al., 1999)	Facilitation of glutamate release and excitation (*via* TRPV1; Marinelli et al., 2003; Melis et al., 2008)
**Non-cannabinoid *N*-acylethanolamines**
Oleoylethanolamide 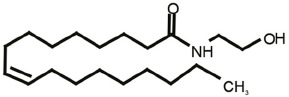	PPARα (Fu et al., 2003)	Phosphorylation and negative modulation of β*-nAChRs (Melis et al., 2008)
Palmitoylethanolamide 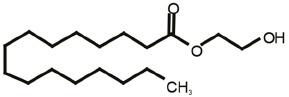	PPARα (Lo Verme et al., 2005)	Phosphorylation and negative modulation of β*-nAChRs (Melis et al., 2008)
***N*-acyl amino acids**
Oleoylglycine 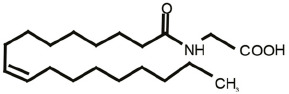	PPARα (Takao et al., 2015; Donvito et al., 2019)	Unknown

*See text for abbreviations*.

Among NAEs, the cannabinoid agonist AEA binds to CB1 and CB2 receptors and transient receptor potential vanilloid type 1 (TRPV1) at physiologically relevant concentrations, the others display an affinity for peroxisome proliferator-activated receptor-α (PPARα; Lo Verme et al., 2005; Hansen, 2010; Petrosino et al., 2010; Pistis and Muntoni, 2017), G protein-coupled receptors such as GPR55 (Baker et al., 2006) and GPR119, and TRPV1 (Piomelli, 2013). *N-*acyl amino acids are less characterized; however, evidence suggests a role for GPR18, GPR55, and GPR92, and PPARα in mediating some of the actions of *N-*oleoylglycine (Burstein, 2018; Donvito et al., 2019), which is one of the most studied among these molecules.

Although several of these molecules were known for decades, physiological activities of NAEs or *N-*acyl amino acids in the CNS and their role in neurological and psychiatric disorders, ranging from substance use disorder, neurodegenerative diseases, epilepsy, and mood disorders (Pistis and Melis, 2010; Melis and Pistis, 2014; Scherma et al., 2016; Pistis and Muntoni, 2017) has been characterized only relatively recently.

## *N-*Acylethanolamines and *N-*Acyl Amino Acids: Synthesis and Catabolism

**Figure 1:**
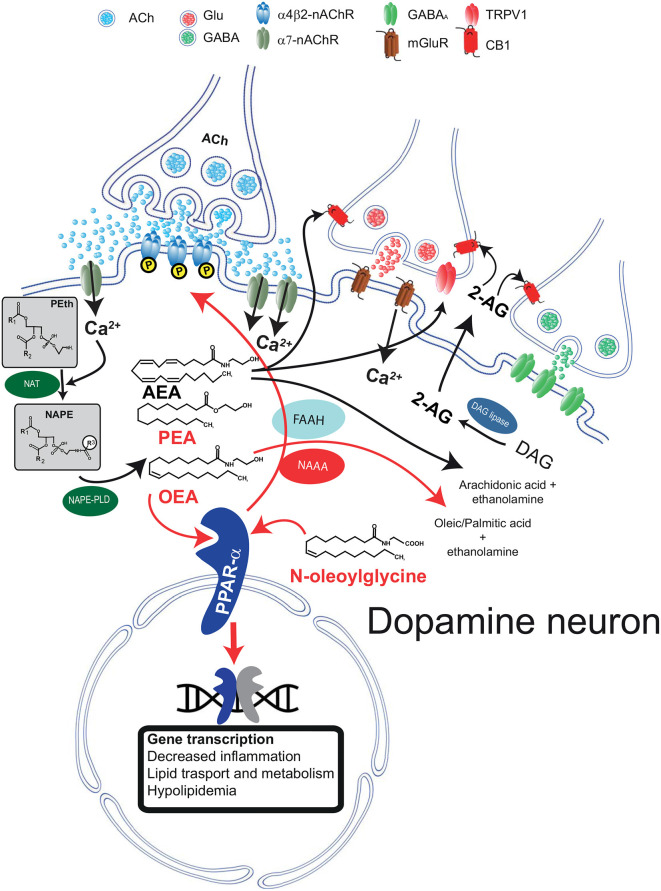
Schematic diagram illustrating the biosynthetic and catabolic pathways for *N-*acylethanolamine (NAE) and canonical endocannabinoid formation and catabolism, and their cellular mechanisms of actions through their receptors. Phosphatidylethanolamine (Peth) is converted into *N-acyl*-phosphatidylethanolamine (NAPE) by *N-*acyltransferase (NAT). Ca^2+^ entry mediated by α7-nAChRs activates NAEs synthesis through the Ca^2+^ dependent NAT. The resulting NAPE is hydrolyzed by NAPE-PLD to the corresponding NAEs anandamide (AEA), oleoyl ethanolamide (OEA), and palmitoylethanolamide (PEA). Activation of PPARα by NAEs results in genomic effects (gene transcription) and in non-genomic actions, such as activation of a tyrosine kinase and phosphorylation of β2^*^nAChRs (i.e., α4β2). Fatty acid amide hydrolase (FAAH) and NAE-hydrolyzing acid amidase (NAAA) are the major inactivating enzymes for OEA, PEA, and AEA and convert them in ethanolamine and corresponding fatty acids (oleic, palmitic, and arachidonic acids, respectively). NAAA preferentially hydrolyzes PEA. *N-*oleoyl glycine is one member of the *N-acyl* amino acid family and is known to activate PPARα. The figure illustrates that AEA and 2-arachidonoylglycerol (2-AG) are produced on demand by NAPE-PLD and DAG lipase, respectively. Raises in intracellular Ca^2+^ can be induced, as in the example, by activation of metabotropic glutamate receptors (mGluR). 2-AG and AEA bind to presynaptic CB1 receptors expressed on GABA and glutamate terminals and depress neurotransmitter release. AEA also activates TRPV1 receptors located on presynaptic glutamatergic terminals. Abbreviations: NAPE-PLD, *N-acyl* phosphatidylethanolamine phospholipase D; DAG, diacylglycerol; MAG, monoacylglycerol; FAAH, fatty acid amide hydrolase; Glu, glutamate; CB1, cannabinoid type-1 receptor; TRPV1, transient receptor potential vanilloid type-1; PPARα, peroxisome proliferator-activated receptor type-α; nAChRs, nicotinic acetylcholine receptors. This figure is adapted, with permission, from Melis and Pistis (2012) and Pistis and Muntoni (2017).

Both AEA and other non-cannabinoid NAEs share both biosynthetic and catabolic pathways. Unlike typical neurotransmitters, their levels are regulated on-demand by enzymes responsible for their synthesis and degradation (Ueda et al., 2010a; Rahman et al., 2014) and not by vesicular release. They are synthesized from membrane-derived *N*-acylphosphatidylethanolamines (NAPEs; Hansen et al., 2000; Okamoto et al., 2004; Hansen, 2010; Ueda et al., 2010b; Rahman et al., 2014). The first step is the generation of the corresponding NAPE by a Ca^2+^-dependent *N*-acyltransferase (NAT; Hansen et al., 2000; Hansen and Diep, 2009); NAPE is then hydrolyzed by NAPE-hydrolyzing phospholipase D (NAPE-PLD) with the generation of NAEs (Rahman et al., 2014; Figure 1).

Very little is known about the biosynthesis of *N-*acyl amino acids, except for *N-acyl* glycines, where it is hypothesized that glycine is directly condensed with the free fatty acid or CoenzymeA derivative acyl moiety by cytochrome C or glycine *NAT*-like 2 and 3 enzymes (Huang et al., 2001; McCue et al., 2008; Waluk et al., 2010; see Burstein, 2018; Battista et al., 2019; for comprehensive reviews of *N-acyl* amino acids).

When catabolized, NAEs and *N-*acyl amino acids are hydrolyzed to free fatty acids and ethanolamine or amino acids (Cravatt et al., 1996; Deutsch et al., 2002; Battista et al., 2019), respectively (Figure 1). This hydrolysis is catalyzed mainly by two major intracellular enzymes, although alternative pathways have been described (Bornheim et al., 1993; Hampson et al., 1995; Ueda et al., 1995; Yu et al., 1997; Kozak et al., 2002). The first enzyme to be characterized is the fatty acid amide hydrolase (FAAH; Cravatt et al., 1996). FAAH hydrolyzes all NAEs and several *N-acyl* amino acids with high efficiency, and it is expressed in many different tissues and cell types, including in the brain. The second major enzyme is the NAE-hydrolyzing acid amidase (NAAA; Tsuboi et al., 2005), which displays a significant preference for unsaturated NAEs such as PEA (Tsuboi et al., 2007). NAAA displays lower expression in the brain, yet significant CNS effects are obtained with selective inhibitors (Sagheddu et al., 2019), suggesting that despite low expression levels, this enzyme exerts physiologicallyrelevant actions by controlling brain levels of NAEs.

Stimuli triggering NAEs’ synthesis vary between AEA and other NAEs. Endocannabinoids like AEA and 2-arachidonoylglycerol (2-AG) are synthesized following activation of metabotropic glutamate, muscarinic, or dopamine D2 receptors (Melis et al., 2004a,b; Kano et al., 2009). Besides the activation of metabotropic receptors, both AEA, 2-AG, and non-cannabinoid NAEs synthesis is initiated by a rise in intracellular Ca^2+^ (Luchicchi and Pistis, 2012; Melis et al., 2013b). The reason for this similarity is presumed to be the Ca^2+^-dependency of the NAT enzyme. In experimental settings, the contribution of specific lipid neuromodulators can be disentangled with pharmacological tools, i.e., selective antagonists at their cellular receptors. Interestingly, both the canonical eCB signaling mediated by AEA/2-AG and the non-canonical OEA/PEA signaling have been characterized in dopamine neurons. However, it is still not clear whether these two separate signaling systems coexist in the same cell. As they are activated by different stimuli, they might fulfill different physiological functions. This scenario is consistent with the idea that NAE signaling displays exquisite anatomical and functional specificity. For example, activation of glutamate afferents in dopamine cells induces synthesis of 2-AG that, *via* CB1 receptors, dampens glutamate release (Melis et al., 2004a). On the other hand, acetylcholine release activates the OEA/PEA signaling (see below; Melis et al., 2010). Thus, both 2-AG and OEA/PEA signaling converge to reduce dopamine cell excitability, contributing to diminishing cellular overdrive induced by excitatory afferents. The functional relevance of these two distinct yet parallel systems needs to be fully understood.

The subcellular localization of NAPE-PLD in the brain is indicative of the possible functional significance of NAEs in the CNS (Cristino et al., 2008; Egertová et al., 2008; Nyilas et al., 2008; Suárez et al., 2008; Reguero et al., 2014; Merrill et al., 2015), as NAPE-PLD mRNA and immunoreactivity are detected both presynaptically and postsynaptically, but with a preferential distribution in postsynaptic dendrites (Reguero et al., 2014). The preferential postsynaptic localization of NAPE-PLD and NAEs affinity to nuclear receptors (see below) indicates that they may act as autocrine or paracrine signals at receptors expressed in the same or neighboring cells.

## *N*-Acylethanolamine Receptors: Peroxisome Proliferator-Activated Receptor-α (PPARα)

PEA, OEA, and *N-*oleoylglycine targets have been identified as the PPAR, and specifically the α-isoform (PPARα). Very little is known about the functional relevance of *N-*acyl amino acids and their receptors such as GPR18, GPR55, or GPR92; this topic is discussed in Burstein (2018) and Battista et al. (2019).

In the brain, considerable evidence suggests that NAEs display activity through PPARα, receptors ubiquitously expressed in the CNS by neurons and glial cells (Braissant et al., 1996; Auboeuf et al., 1997; Mandard et al., 2004; Moreno et al., 2004; Galan-Rodriguez et al., 2009; Fidaleo et al., 2014).

PPARs belong to the large superfamily of transcription factors, composed of three isotypes: PPARα, PPARγ and PPARβ/δ (Germain et al., 2006). The large ligand-binding site of PPARs can accommodate a variety of diverse lipophilic endogenous ligands and synthetic agonists, including fibrates, clinically approved for decades for the treatment of hypertriglyceridemia. Hence, PPARα is a transcriptional regulator of genes involved in peroxisomal and mitochondrial β-oxidation, and fatty acid transport (Xu et al., 2002). PPARα is also engaged in the anti-inflammatory response, as it negatively regulates pro-inflammatory pathways and signals involved in the acute phase response in models of systemic inflammation (Berger and Moller, 2002; Gervois et al., 2004; Moreno et al., 2004; Glass and Ogawa, 2006; Bensinger and Tontonoz, 2008).

## PPARα and Dopamine Cells

The anatomical and functional segregation between cannabinoid and non-cannabinoid NAEs in their cellular effects is evident in dopamine cells, where these two signaling systems have been characterized. Dopamine cells synthesize and release eCBs following activation of metabotropic receptors, membrane depolarization, and Ca^2+^ entry (Melis et al., 2004a,b). Released eCBs bind to presynaptic CB1 receptors expressed by GABA and glutamate terminals (Melis et al., 2004b, 2013a, 2014; Pistis et al., 2004; Figure 1). The functional relevance of eCB signaling is reviewed elsewhere (Melis and Pistis, 2007, 2012; Melis et al., 2012). Here, it suffices to say that eCBs sculpt short- and long-term forms of synaptic plasticity and fine-tune firing activity of dopamine cells, specifically in tasks where these neurons are engaged, such as reward and motivation.

A different scenario is emerging when NAEs are concerned. In dopamine cells, NAE synthesis is triggered by enhanced nicotinic cholinergic transmission, and the switch was identified as the low-affinity extrasynaptic α7 nicotinic acetylcholine receptor (α7-nAChRs; Jones, 2004; Yang et al., 2009; Figure 1). The interpretation for this finding is that this receptor, expressed in sites distant from cholinergic axon terminals (Jones, 2004), is a sensor for an intense cholinergic drive, being activated by acetylcholine (Yang et al., 2009) spilled over from cholinergic synapses impinging onto dopamine cells. α7-nAChRs are permeable to Ca^2+^ ions, and their activation by acetylcholine or exogenous ligands (i.e., nicotine) evokes an increase in Ca^2+^ permeability and a rise in intracellular Ca^2+^, which is necessary for the activity of the Ca^2+^-dependent NAT isoform (Ogura et al., 2016; Hussain et al., 2018). The result is a rise in levels of PEA and OEA, which, differently from eCBs, bind to intracellular receptors within the dopamine cell, acting as autocrine-like signals (Melis et al., 2013b; Figure 1) in a fashion similar to other neuromodulators, such as neurotrophic factors (Herrmann and Broihier, 2018). Other laboratories have confirmed the interaction between α7-nAChRs and PPARα in different settings: Donvito et al. (2017) observed that the antinociceptive effects of α7-nAChR agonists were mediated by a rise in PEA and activation of PPARα and Jackson et al. (2017) confirmed that PPARα is involved in the effects mediated by α7-nAChR agonists in nicotine dependence.

These effects have the potential to regulate synaptic functions. Our studies show that PPARα activation in VTA dopamine cells triggers, *via* endogenous hydrogen peroxide and consequent activation of tyrosine kinase(s) (Melis et al., 2008, 2010), phosphorylation of the β2 subunits of the nAChRs (Melis et al., 2013b). Phosphorylation of nAChR subunits is an efficient way to regulate receptor functions by inducing a faster desensitization rate or a downregulation *via* internalization (Huganir and Greengard, 1990). Cholinergic inputs control firing rate and burst firing of midbrain dopamine cells *via* nAChRs (Mameli-Engvall et al., 2006), thus the functional regulation of β2 subunits, that together with α4 are the main nAChR subunits expressed by VTA DA neurons (Champtiaux et al., 2003), might prove useful in nicotine addiction (see below) dopamine-related neurological or psychiatric disorders.

While modulation of nAChRs by PPARα is one of the likely mechanisms by which these nuclear receptors acutely control dopaminergic transmission, we must take into account that genomic effects might also be highly relevant, e.g., anti-inflammatory effects. This is particularly important in psychiatric and neurological disorders when altered synaptic transmission and neuroinflammation interact to generate pathological phenotypes.

As non-cannabinoid NAEs are engaged by dopamine cells as an autocrine-like signal through PPARα to regulate afferent projections and their own pattern of activity, it is not surprising that these lipid neuromodulators might play a major role in substance use disorders (SUD).

An extensive literature substantiates the role of the dopamine system in addiction and SUD. Dopamine facilitates the development of long-lasting forms of synaptic adaptations that determine the effectiveness of reward and reward predictors to control subsequent seeking behavior (Wise and Robble, 2020). Among several aspects of dopamine function related to addiction, the phasic firing of dopamine neurons sculpts learning processes, particularly when learning is associated with rewarding stimuli or its opposite, aversion (Wise and Robble, 2020).

Evidence is accumulating that metabolic enzymes and receptors of these eCB-like signals might be a target for medications in SUD, and specifically alcoholism or nicotine dependence. In contrast, evidence linking them to psychostimulant or opioid use disorders is still very limited.

## Role of NAEs and PPARα in Alcohol Use Disorder

It is well established that the eCB system in dopamine regions contributes to the motivation to consume alcohol. Evidence derives, among others, from the observation that the innate extent of susceptibility to alcohol use disorders (AUD) depends on increased eCB levels within mesolimbic dopamine regions (Basavarajappa et al., 2006; Sagheddu and Melis, 2015), and that administration of CB1 receptor antagonists reduces alcohol drinking in animal models of alcoholism (Colombo et al., 1998). Consistently, alcohol self-administration is controlled by CB1 receptors in the VTA-NAc circuit of alcohol-preferring rats (Malinen and Hyytia, 2008), and Sardinian alcohol-preferring rats show enhanced eCB-mediated synaptic plasticity in the VTA when compared with Sardinian non preferring rats as controls (Melis et al., 2014).

Evidence is recently accumulating on non-cannabinoid NAEs’ contribution to AUD (Orio et al., 2019). OEA has been shown to reduce behavioral expression of withdrawal, such as manifest signs of distress and alcohol-seeking (Bilbao et al., 2016). In rats, this is associated with the molecular effects of OEA, which counteracts alcohol-induced glial and neuronal alterations in brain regions processing drug reward (Rivera et al., 2019). Being antioxidant, anti-inflammatory, and neuroprotective, OEA, and PEA are considered molecules with therapeutic potential in comorbid disorders, including depression and anxiety in AUD (Pistis and Muntoni, 2017).

The role of NAEs in alcohol dependence has been extensively explored by studying the catabolic enzyme FAAH, both in rodents and humans. Several studies stress out the importance of FAAH genetic variants (Zhou et al., 2016; Sloan et al., 2018), or its enzymatic functionality as a factor contributing to the severity of the pathology. Recently, a PET scan study for a FAAH radiotracer was conducted in the brain of AUD patients during early abstinence. It showed transiently reduced FAAH levels, while its substrates AEA, OEA, and *N-*docosahexaenoyl ethanolamide (DEA) were elevated in the plasma (Best et al., 2020). Low FAAH levels are related to drinking behaviors and increased preference for alcohol, as demonstrated in several studies using animal models with genetic deletions (Basavarajappa et al., 2006; Blednov et al., 2006; Vinod et al., 2008; Pavón et al., 2018); or following pharmacological manipulation (Zhou et al., 2017).

PPARα is upstream of diverse genes that are modulated by ethanol or involved in ethanol-induced effects (Ferguson et al., 2014). Preclinical studies showed that modulation of PPARα by the synthetic agonist fenofibrate reduced motivational and reinforcing properties of ethanol, as measured by voluntary drinking in mice (Ferguson et al., 2014; Blednov et al., 2016) and rats (Karahanian et al., 2015); and corroborated by the self-administration paradigm in rats (Haile and Kosten, 2017). Considering that fibrates are approved for medical conditions, these studies suggest that regulation of PPARα deserves further clinical investigation in AUD, as recently detailed elsewhere (Karahanian et al., 2015; Matheson and Le Foll, 2020). Nonetheless, there are no pending clinical trials to date. An interesting pharmacological approach takes advantage of combining drugs acting at PPARα and other receptors. PPARα/γ dual agonists have proven to reduce alcohol consumption in both mice (Blednov et al., 2015) and rats (Alen et al., 2018). Dual CB1 antagonist/PPARα agonist reduced voluntary ethanol intake and self-administration in rat models of AUD (Alen et al., 2018), arising a promising step forward to the safe pharmacological manipulation of the eCB system.

## Role of NAEs and PPARα in Addiction: Nicotine Dependence

Tobacco use is associated with high morbidity and mortality, it being the most preventable cause of death in the world (World Health Organization, 2019). Nicotine, the main psychoactive component in tobacco, is one of the most addictive substances (Picciotto and Mineur, 2014) and exerts its effects through nAChRs.

Both tobacco smoke and nicotine can affect the eCB system. Tobacco smoke alters FAAH, NAPE-PLD, and MAGL levels in the striatum (Torres et al., 2019), while nicotine modifies eCB signaling according to the administration protocol. For instance, nicotine self-administration decreased OEA and increased AEA and 2-AG levels, while nicotine infusion, as well as mecamylamine-induced nicotine withdrawal, only increased 2-AG levels (Buczynski et al., 2013; Saravia et al., 2017). A critical role for 2-AG was also demonstrated in nicotine reinforcement (Buczynski et al., 2016) and in nicotine-induced dopamine release (Cheer et al., 2007). The overlap of nAChRs and the receptors of the eCB system in brain areas critical to nicotine effects, such as the mesolimbic system, shows that the eCB system plays an important role in nicotine dependence (Gamaleddin et al., 2015).

The involvement of the eCB system in nicotine dependence was demonstrated by the effect of FAAH inhibitors. FAAH inhibitors suppress many reward-related effects of nicotine in rats and non-human primates, such as nicotine self-administration and reinstatement of nicotine seeking (Scherma et al., 2008; Forget et al., 2009; Gamaleddin et al., 2015; Justinova et al., 2015); nicotine-induced excitation of dopamine neurons in the VTA (Melis et al., 2008), and dopamine release (Scherma et al., 2008). Importantly, the involvement of both CB1 and PPARα receptors was reported.

Besides the effect of the major eCBs, there is increasing evidence of the involvement of PEA and OEA in nicotine addiction, as they have a crucial role as endogenous modulators of cholinergic transmission (Melis et al., 2013b). Moreover, they also inhibit nicotine addictive behaviors, changing dopamine cell excitability. All these actions are due to the activation of PPARα, rather than CB1 receptor (Melis et al., 2008), being dependent on PEA and OEA synthesis as a result of activation of the cholinergic system, *via* α7-nAChRs, and subsequent increase of intracellular Ca^2+^ (Melis et al., 2013b; Figure 1). Using *in vivo* and *in vitro* strategies, Melis et al. (2010) confirmed that the β2 subunit is crucial for PPARα effects, as re-expression of β2 receptors in VTA dopamine cells in β2 knockout mice was sufficient to rescue PPARα effects. Consistently, *N-*oleoyl glycine was shown by Donvito et al. (2019) to counteract several effects related to nicotine reward and dependence, including the withdrawal syndrome, with a PPARα-dependent mechanism. It is not known if this *N-acyl* amino acid is synthesized in dopamine cells and acts as an endogenous neuromodulator in a similar fashion of other NAEs with a dopamine moiety such as *N-*arachidonoyldopamine or *N-*oleoyldopamine (Ferreira et al., 2009; Sergeeva et al., 2017), so its role in the modulation in dopamine-mediated behaviors such as SUD are not clear yet.

Based on the mechanisms described, the suppression of nicotine-induced responses of dopamine neurons by PPARα agonists raised the interest on these ligands as a promising strategy to prevent nicotine relapse (Melis and Pistis, 2014; Matheson and Le Foll, 2020). To date, two clinical studies investigated the effects of PPARα agonists—gemfibrozil and fenofibrate—in smoking cessation, as a drug repositioning strategy. The authors did not observe beneficial effects of gemfibrozil or fenofibrate in treatment-seeking smokers (Perkins et al., 2016; Gendy et al., 2018). It must be pointed out that these disappointing results might be due to the low agonist potency and limited brain permeability in humans of clinically approved fibrates, which might result in low brain concentrations of these drugs, insufficient to achieve an optimal PPARα activation in the CNS. Indeed, doses of fenofibrate higher than those tested in the studies mentioned above were beneficial in a form of epilepsy induced by a gain of function of nAChRs, the sleep-related hyper motor epilepsy (SHE; formerly termed nocturnal frontal lobe epilepsy, NFLE; Puligheddu et al., 2017).

A way to circumvent the limited brain permeability of fibrates is to increase brain levels of endogenous PPARα agonists, such as PEA and OEA. The recent development of brain-permeant selective NAAA inhibitors offers the advantage to modulate levels of PEA and OEA selectively, and not AEA, therefore concurrently limiting psychiatric side effects due to eCB-CB1 alteration. Similar to direct PPARα agonists, also NAAA inhibitors display potential as anti-smoking medications, as they block nicotine-induced excitation of dopamine cells, dopamine elevations in the nucleus accumbens, and conditioned place preference in a PPARα-dependent manner (Sagheddu et al., 2019).

## Concluding Remarks

The expanded eCB system, the “endocannabinoidome,” is a hotbed for a large number of lipid signaling molecules, enzymes, and receptors and represents a Pandora’s box for drug discovery (Cristino et al., 2020).

This review article summarizes evidence suggesting that NAE/PPARα signaling shows promise as a target in the treatment of SUD, particularly alcohol and nicotine use disorder. A parsimonious unifying hypothesis for this effect is NAE/PPARα’s ability to modulate dopamine cell activity by specifically dampening stress-evoked excitatory drive from cholinergic afferents on VTA dopamine cells. Hence, a heightened cholinergic transmission has long been postulated to contribute to detrimental effects induced by stress, such as depression (Janowsky et al., 1972) and drug addiction (Morel et al., 2018; Shinohara et al., 2019). Consistently, selective inhibition of cholinergic neurons in the laterodorsal tegmentum, which provides a major cholinergic input to dopamine cells, prevents stress-induced cellular adaptations within VTA dopamine cells and the appearance of anhedonia and social withdrawal (Fernandez et al., 2018). Additionally, PPARα activation attenuates effects induced by stress (Scheggi et al., 2016; Ni et al., 2018; Song et al., 2018; Locci and Pinna, 2019), corroborating the idea that the interplay between stress, cholinergic inputs, dopamine neurons, and PPARα signaling might play a pivotal role in the potential favorable effects of NAEs in SUD.

SUD represents an unmet clinical need, with drugs currently in use that show limited efficacy or untoward side effects. Indeed, results reported for members of the NAE and *N-*acyl amino acid family suggest that analogs of these lipid neuromodulators could become potential drug candidates.

## Author Contributions

CS, LT, TM, and MP wrote the manuscript. All authors contributed to the article and approved the submitted version.

## Conflict of Interest

The authors declare that the research was conducted in the absence of any commercial or financial relationships that could be construed as a potential conflict of interest.
